# Topological analysis of the *Escherichia coli* WcaJ protein reveals a new conserved configuration for the polyisoprenyl-phosphate hexose-1-phosphate transferase family

**DOI:** 10.1038/srep09178

**Published:** 2015-03-17

**Authors:** Sarah E. Furlong, Amy Ford, Lorena Albarnez-Rodriguez, Miguel A. Valvano

**Affiliations:** 1 Centre for Human Immunology, Department of Microbiology and Immunology, University of Western Ontario, London, Ontario, N6A 5C1, Canada; 2 Centre for Infection and Immunity, Queen's University Belfast, Belfast, United Kingdom, BT9 7AE

## Abstract

WcaJ is an *Escherichia coli* membrane enzyme catalysing the biosynthesis of undecaprenyl-diphosphate-glucose, the first step in the assembly of colanic acid exopolysaccharide. WcaJ belongs to a large family of polyisoprenyl-phosphate hexose-1-phosphate transferases (PHPTs) sharing a similar predicted topology consisting of an N-terminal domain containing four transmembrane helices (TMHs), a large central periplasmic loop, and a C-terminal domain containing the fifth TMH (TMH-V) and a cytosolic tail. However, the topology of PHPTs has not been experimentally validated. Here, we investigated the topology of WcaJ using a combination of LacZ/PhoA reporter fusions and sulfhydryl labelling by PEGylation of novel cysteine residues introduced into a cysteine-less WcaJ. The results showed that the large central loop and the C-terminal tail both reside in the cytoplasm and are separated by TMH-V, which does not fully span the membrane, likely forming a "hairpin" structure. Modelling of TMH-V revealed that a highly conserved proline might contribute to a helix-break-helix structure in all PHPT members. Bioinformatic analyses show that all of these features are conserved in PHPT homologues from Gram-negative and Gram-positive bacteria. Our data demonstrate a novel topological configuration for PHPTs, which is proposed as a signature for all members of this enzyme family.

The biosynthesis of bacterial glycoconjugates such as cell wall peptidoglycan, exopolysaccharides, and the O antigen moiety of the lipopolysaccharide starts by the formation of a phosphoanhydride linkage between undecaprenyl pyrophosphate (Und-P) and a sugar-1-phosphate donated from a sugar nucleoside diphosphate^1^. Two distinct families of membrane enzymes, termed polyisoprenyl-phosphate N-acetylaminosugar-1-phosphate transferases and polyisoprenyl-phosphate hexose-1-phosphate transferases (PHPT), catalyse this reaction^2^,^3^. These enzymes are indispensable for lipid-linked glycan biosynthesis because they connect soluble cytosolic components (sugar phosphates donated as nucleotide sugars) with membrane-bound Und-P lipid carrier.

Unlike N-acetylaminosugar-1-phosphate transferases, PHPTs have no known eukaryotic homologues^2^. PHPTs initiate the biosynthesis of capsule^4^,^5^, O antigen^6^, and glycans attached to bacterial glycoproteins^7^. Most family members share similar predicted topology to that of *Salmonella enterica* WbaP, a prototypic PHPT that consists of five predicted transmembrane helices (TMHs), a large periplasmic loop, and an extended C-terminal region (Fig. 1a)^6^,^8^,^9^. While the predicted N-terminal and periplasmic domains are not always present in proteins of the PHPT family, the TMH-V and the C-terminal domain are conserved in all family members, and sufficient for activity in those proteins that naturally possess that "simpler" structure, as well as in truncated versions of the more complex family members^6^,^8^,^10^. The function of the N-terminal and large periplasmic loop domains is unclear. Deletion or overexpression of the predicted periplasmic loop of WbaP in *S. enterica* affects the distribution of O-antigen chain length and deletion mutant proteins show reduced *in vitro* transferase activity^6^. In the Gram-positive bacterium *S. pneumoniae*, the predicted extracellular loop of the glucosyltransferase Cps2E (corresponding to the periplasmic domain in PHPTs from Gram-negative bacteria) is important in modulating polymer assembly and the rate of the initiation reaction^4^,^11^. Therefore, the large periplasmic/extracellular domain of PHPT proteins could regulate the catalytic region located on the C-terminal domain^3^.

Unfortunately, much of the current understanding of PHPT function relies on a predicted topological model of the protein that has not been experimentally validated. The predicted topology of WbaP was first proposed by Valvano^2^ based on combined bioinformatic data from DAS and TMHMM programs^12^,^13^, which indicated two large soluble regions separated by one TMH. The DAS program does not provide topological information for the location of soluble domains, while TMHMM suggested that the C-terminal soluble domain was in the periplasmic space (Fig. 1b). Since this prediction did not agree with experimental data indicating that the C-terminal half of WbaP is required for the transferase function^9^, the C-terminal region of WbaP was assigned to the cytosolic space, where sugar nucleoside diphosphate precursors become available. This assignment was subsequently validated experimentally by protease accessibility experiments and microscopy using recombinant WbaP C-terminally fused to the green fluorescent protein, a reporter for cytoplasmic location of membrane protein segments^8^. However, *in silico* predictions of PHPT proteins give inconsistent results depending on the program used (Fig. 1b). Major areas of disagreement in the predictions are the number of TMHs (5 to 7) and the subcellular localization of the periplasmic/extracellular region and the C-terminal tails (Fig. 1b). Protease accessibility experiments in WbaP showed no cleavage in the predicted large periplasmic region despite the presence of multiple trypsin cleavage sites, suggesting that this domain may not be located in the periplasm^8^. In contrast, using LacZ and PhoA fusions combined with secondary structure predictions, Videira *et al.*^14^ proposed that BceB, a PHPT enzyme that initiates the biosynthesis of the cepacian exopolysaccharide in *Burkholderia cenocepacia*, consists of seven TMHs. This model also predicts a novel TMH adjacent to TMH-V and another TMH near the C-terminus that would place the C-terminal end of the protein on the periplasmic space (Fig. 1c)^14^. The inconsistencies between *in silico* predictions and limited experimental data^8^,^14^ underscore the need to carefully address the topology of PHPTs.

In this work, we utilized WcaJ, a model PHPT that shares similar features as WbaP^5^. WcaJ is an integral membrane protein in *Escherichia coli* K-12 and other *Enterobacteriaceae,* which catalyses the transfer of glucose-1-phosphate from UDP-glucose (UDP-Glc) to Und-P resulting in the formation of Und-P-P-Glc. This is the first step in the biosynthesis of colanic acid (CA) capsule or M antigen^5^,^15^. CA is a high-molecular weight, extracellular polysaccharide required for biofilm formation, resistance to desiccation, and cell stress responses^16^,^17^. CA is loosely connected with the outer membrane, but under specific conditions can also be attached to the lipopolysaccharide (LPS) core, resulting in M_LPS_^18^. The production of CA is regulated by the *rcs* (regulator of capsule biosynthesis) genes *rcsA*, *rcsB*, and *rcsC*. RcsA and RcsB proteins are positive transcriptional regulators and are required for maximal CA production^19^.

Here, we report a detailed topological analysis of WcaJ using two methods: reporter LacZ/PhoA fusions and sulfhydryl labelling by PEGylation of novel cysteine residues that are introduced into a cysteine-less (Cys-less) WcaJ protein. Our results reveal that WcaJ comprises 4 canonical TMHs and a TMH-V that does not fully span the membrane, likely forming a hairpin. This topological arrangement places both the adjacent central loop and C-terminal domain towards the cytoplasm. The new conserved topological configuration for WcaJ is proposed as a signature for all PHPTs, and provides a framework to better understand the structure-function of this important membrane enzyme family.

## Results and Discussion

### The C-terminus of the *E. coli* WcaJ is sufficient for enzymatic activity *in vivo*

Before using WcaJ as a model PHPT for topological analysis, we determined if its C-terminal domain was functionally comparable to the WbaP C-terminus^10^. Full-length _FLAG_WcaJ (53 kDa) and _FLAG_WcaJ_CT_ (25 kDa)_,_ a truncated version comprising residues E252-Y464, which is analogous to WbaP_CT_ (F258-Y476)^6^,^8^, were constructed and expressed from an arabinose inducible promoter (Fig. 2a). _FLAG_WcaJ migrates by SDS-PAGE with an apparent mass of ~48 kDa. A larger band of ~120 kDa (Fig. 2a, double asterisk) reacting with the anti-FLAG antibody was interpreted as an oligomeric form of WcaJ. Anomalous gel migration and oligomerization are commonly seen with membrane proteins^6^,^20^,^21^, particularly in those with high isoelectric points^22^ (the theoretical pI for WcaJ is 9.2), since the mild denaturing conditions used for the preparation of the cell lysates is insufficient to disperse protein oligomers. Plasmids encoding _FLAG_WcaJ and _FLAG_WcaJ_CT_ (pLA3 and pLA5, respectively) were introduced by transformation into *E. coli* Δ*wcaJ* also containing a plasmid expressing RcsA (pWQ499), the positive transcriptional regulator of the CA biosynthesis gene cluster^23^. In excess of RcsA, CA overproduction generates a highly mucoid colony phenotype^23^. Expression of _FLAG_WcaJ_CT_ and _FLAG_WcaJ in Δ*wcaJ*(pWQ499) restored the mucoid phenotype to the same levels as the parental strain, while no CA was observed in bacteria transformed with the vector pBADNTF (Fig. 2b), demonstrating that the WcaJ C-terminal domain is sufficient for enzymatic activity, as in the other PHPT family members^5^,^10^.

### Topological analysis of WcaJ using LacZ and PhoA reporter fusions reveals an unexpected membrane topology

**Figure 1:**
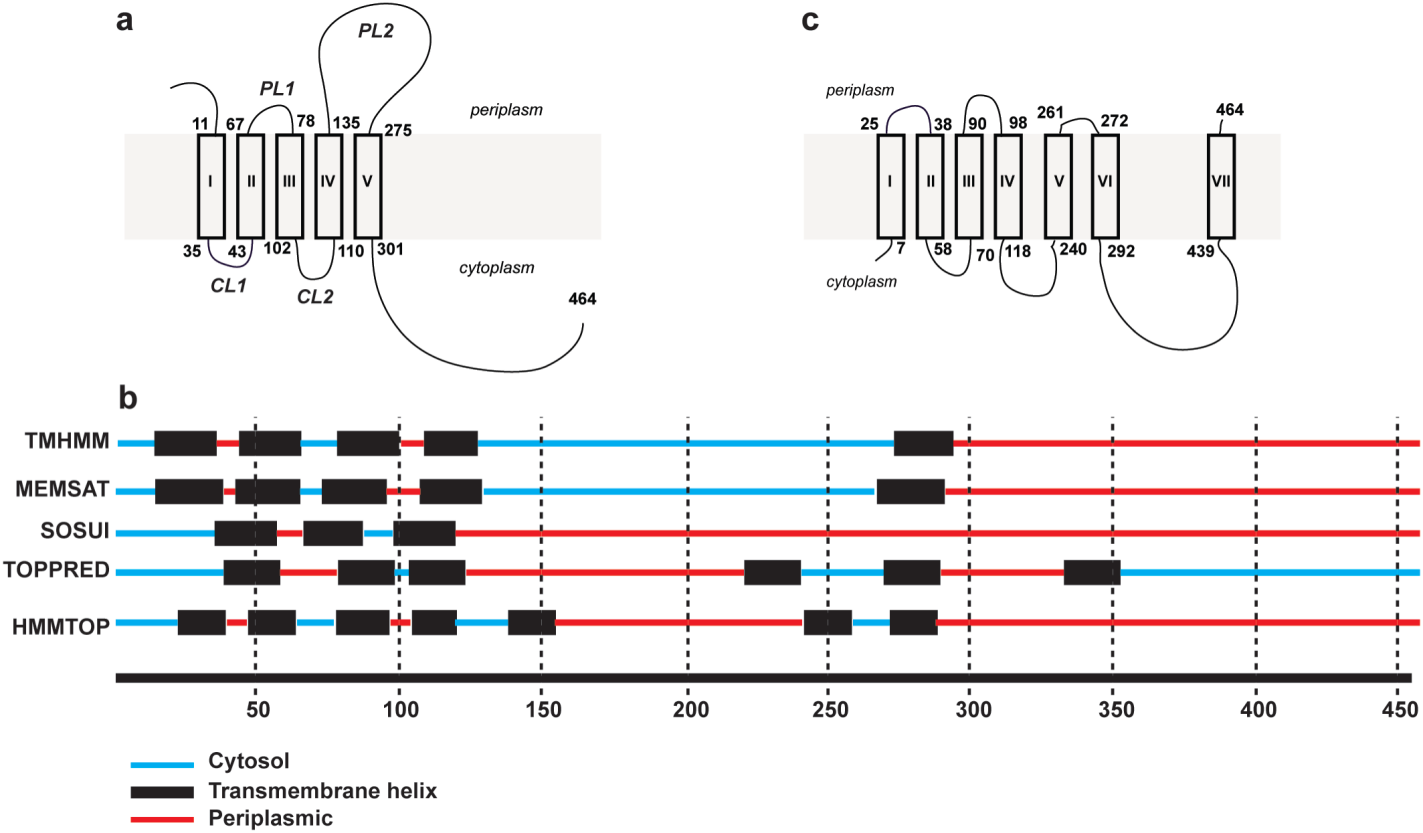
Topologies of PHPT proteins based on bioinformatic predictions. (a) Predicted model for WcaJ from *E. coli* W3110 that is similar to that of WbaP^2^,^6^, as explained in the Introduction. Numbers next to each predicted transmembrane helix (TMH) indicate the position of amino acid residues at the predicted boundaries between soluble and membrane embedded regions. TMHs are indicated by roman numerals. PL, periplasmic loop; CL, cytoplasmic loop. (b) Graphical representation of the topological predictions by various programs. The numbers indicate the position of the amino acid residues in WcaJ (464 amino acids). The location of soluble segments (cytosolic or periplasmic) and the positions of predicted TMHs are indicated. (c) Predicted model for BceB from *B. cenocepacia* IST432, a WcaJ homologue, which was derived from TMH predictions, experimental data, and refined based on hydrophobicity and conformational parameters for beta turns^14^. Numbers next to each TMH indicate the position of amino acid residues at the predicted boundaries between soluble and membrane embedded regions. TMHs are indicated by roman numerals.

**Figure 2:**
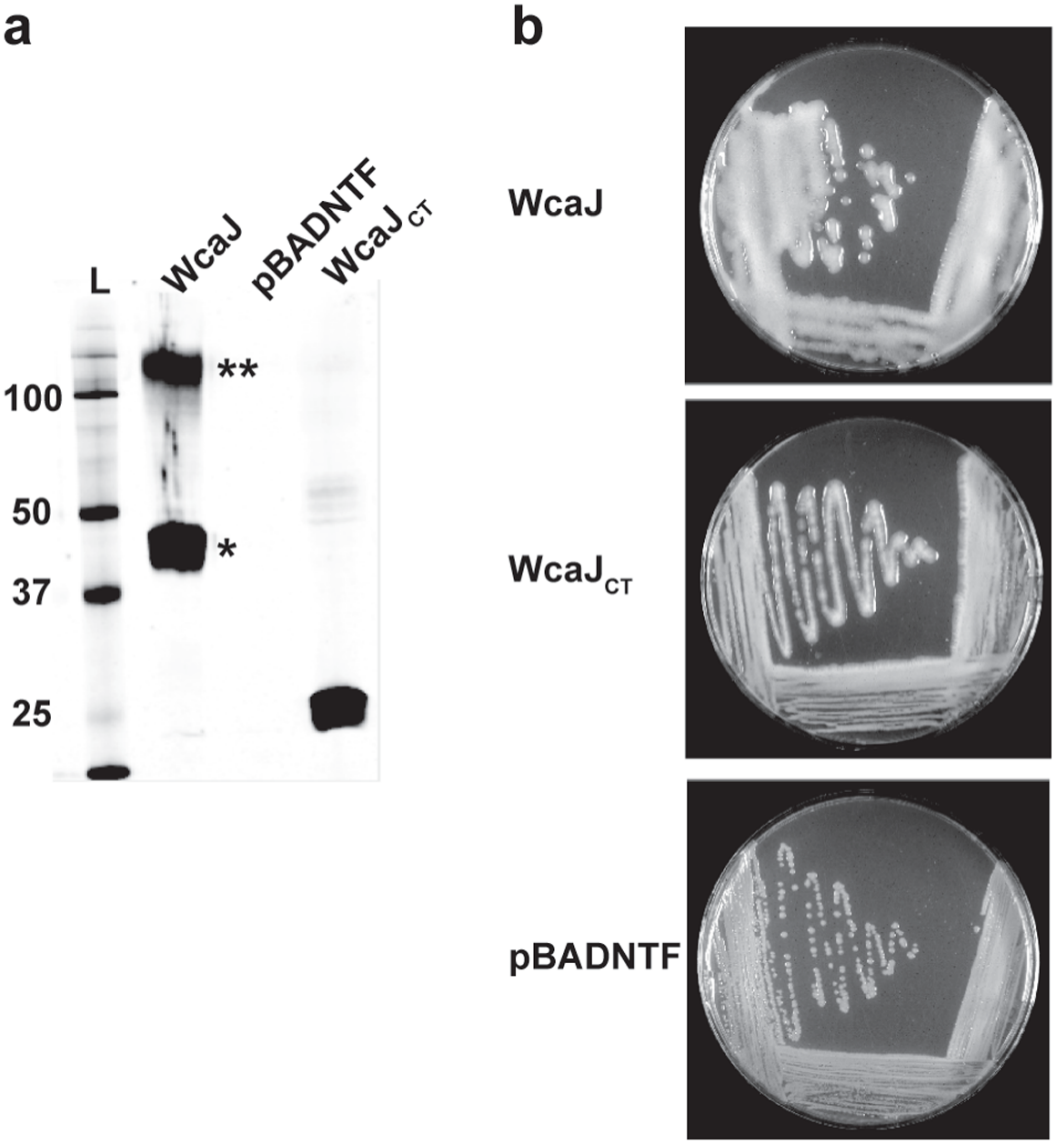
(a) Membrane protein expression of WcaJ and WcaJ_CT_. Both proteins were engineered as N-terminal FLAG-tagged proteins and expressed from the arabinose-inducible vector pBADNTF (see Supplementary Table S2). Total membranes were isolated from *E. coli* DH5α expressing the constructs, separated by electrophoresis in 14% SDS-polyacrylamide gels, and detected by immunoblotting using 1:10,000 dilution of anti-FLAG M2 monoclonal antibody as indicated in Methods. * and ** indicate the position of monomeric WcaJ and oligomeric forms of WcaJ, respectively. L, molecular mass protein ladder (b) Complementation of colanic acid production in *E. coli* XBF1(pWQ499). Bacteria expressing the WcaJ constructs or containing the vector control were plated on LB agar supplemented with antibiotics, as needed, and 0.2% (w/v) arabinose. Colonies were evaluated for mucoid phenotype after 24 h at room temperature.

**Figure 3:**
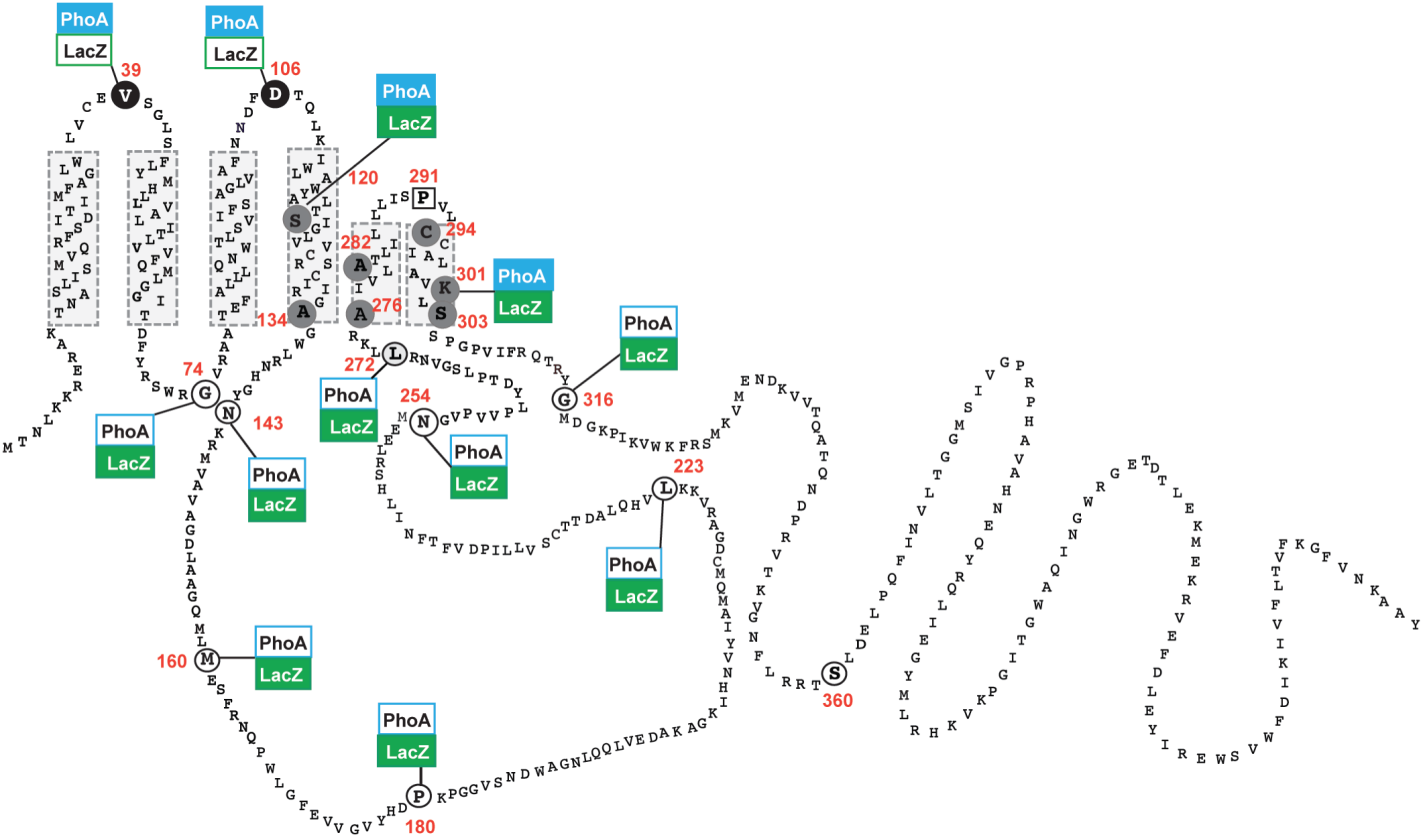
Refined topological model of the *E. coli* WcaJ based on reporter fusions and substituted cysteine labelling with PEG-Mal. Blue coloured boxes indicate positive PhoA results and green coloured boxes indicate positive LacZ results. White coloured boxes indicate negative reporter fusion results. Residues indicated in circles were replaced by cysteine in the WcaJ_Cysless_ version of the protein. Residues that were labelled with PEG-Mal denoting a periplasmic location are depicted with black background. Residues indicated in grey are those only detected by PEG-Mal after SDS solubilization, indicating they are buried in the membrane bilayer. Residues exposed to the cytosol are depicted in white background. The square indicates the position of the conserved P291, which could not be labelled with PEG-Mal under any of the treatments employed including SDS solubilization of membrane fractions.

To experimentally assess the membrane topology of WcaJ (Fig. 1) we employed two complementary approaches: a dual reporter strategy and the substituted cysteine accessibility method (SCAM). The dual reporter fusion strategy was based on PhoA and LacZ reporters that were C-terminally fused to _FLAG_WcaJ truncations and expressed in *E. coli* strains CC118 (Δ*phoA*) and DH5α (*lacZ*ΔM15), respectively. Colonies were examined for blue or white phenotypes on LB-agar plates supplemented with the chromogenic substrates 5-bromo-4-chloro-3-indolyl-phosphate (XP) and 5-bromo-4-chloro-3-indolyl-β-d-galactopyranoside (XGal), as appropriate and the *in vitro* alkaline phosphatase and β-galactosidase activities were measured as Miller units^24^ (Table 1). PhoA is only active in the periplasm, as it requires an oxidizing environment to form disulphide bonds^25^,^26^. Conversely, LacZ functions in the cytosol. The results were consistent with the topological model shown in Fig. 3. _FLAG_WcaJ-LacZ fusions at V39 and D106, which reside in predicted cytoplasmic loops 1 and 2 (Fig. 1a), did not exhibit β-galactosidase activity and the colonies containing these constructs remained white (Table 1)*.* On the contrary, the corresponding _FLAG_WcaJ-PhoA fusions at the same positions yielded strongly blue colonies in XP and positive alkaline phosphatase activity (2112 ± 95 and 938 ± 70 Miller units, respectively; Table 1), indicating that these residues are exposed to the periplasmic space. The LacZ fusion to G74, a residue predicted to be in periplasmic loop 1 (Fig. 1a), resulted in low β-galactosidase activity, but yielded blue colonies on XGal plates while the corresponding PhoA fusion gave no colour on XP plates. This result supports the conclusion that G74 is exposed to the cytosolic space (see below). Furthermore, LacZ fusions at residues in the predicted periplasmic loop II (N143, M160, P180, L223, N254, and L272) gave blue colonies on X-Gal plates and half of them showed strong *in* *vitro* β-galactosidase activity (Fig. 3 and Table 1). None of the corresponding PhoA fusions demonstrated blue colour in XP plates and had very low or no detectable alkaline phosphatase activity (Table 1). These results demonstrate that the large soluble domain of WcaJ predicted to be exposed to the periplasm (Fig. 1a) faces instead the cytoplasmic compartment (Fig. 3).

LacZ and PhoA fusions at S120 and K301 gave inconclusive results, as bacteria expressing these constructs produced blue colonies when plated on XGal or XP plates (Table 1). It has been reported that LacZ and PhoA fusions to amino acid residues located on TMHs can both be functional, failing to provide accurate topological information^27^,^28^. Therefore, we tentatively assigned S120 within TMH-IV and K301 to TMH-V (Fig. 3, see text below). In contrast, the fusions at G316, which is expected to be part of the functional C-terminal domain of WcaJ, gave results consistent with its predicted cytosolic location (Fig. 3 and Table 1). Immunoblotting was performed on total membrane preparations of CC118 cells expressing _FLAG_WcaJ-PhoA or DH5α cells expressing _FLAG_WcaJ-LacZ fusion proteins. All of the fusion proteins were detectable by immunoblot, albeit at different expression levels, suggesting differences in protein stability depending on the fusion joint (Supplemental Figures S1 and S2 online). Together, the reporter fusion data suggest the large soluble loop between TMH-IV and -V is cytoplasmic.

**Figure 4:**
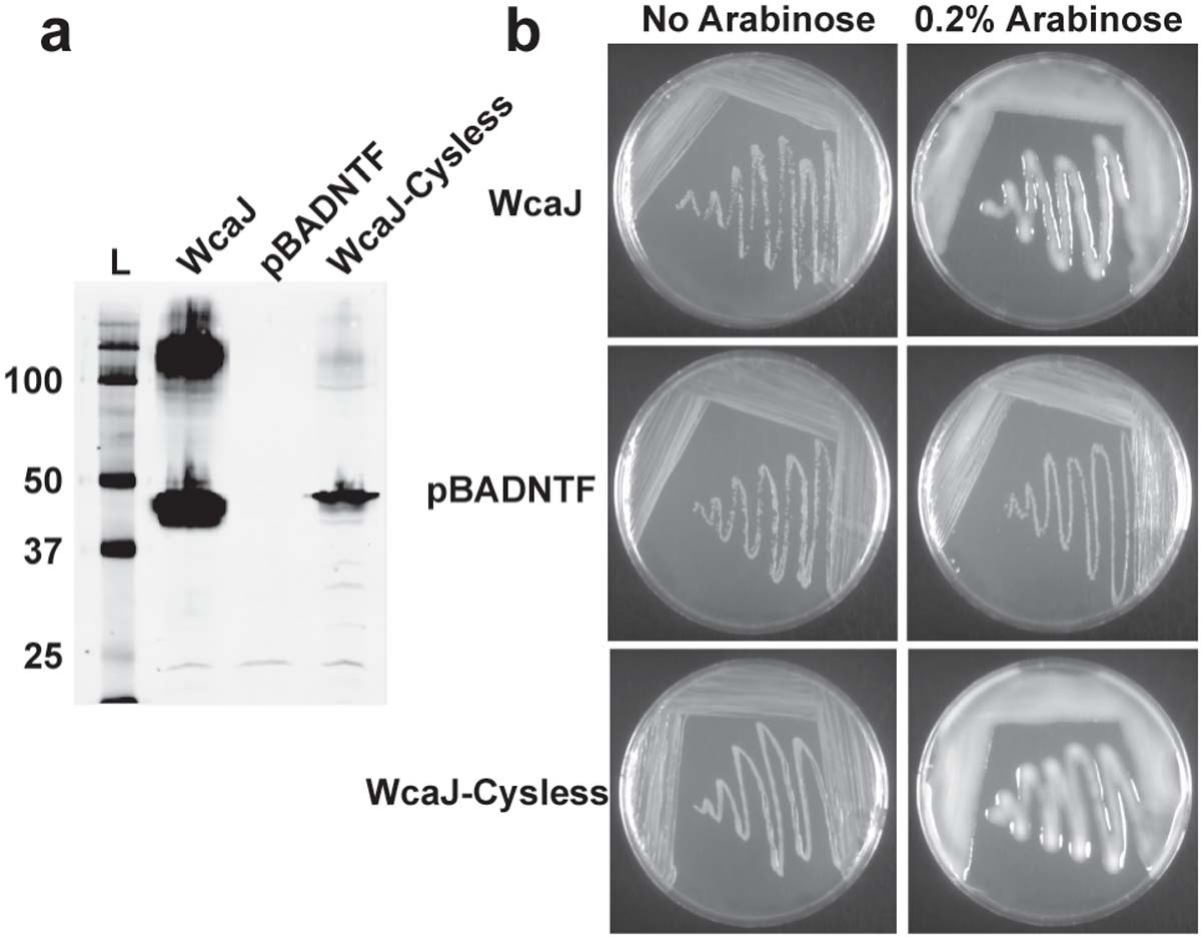
(a) Membrane protein expression of WcaJ_Cysless_. Ten μg of total membrane preparations from *E. coli* DH5α cells expressing WcaJ (pLA3) and WcaJ_CysLess_ (pSEF102), both engineered with an N-terminal FLAG tag, were separated by 14% SDS-PAGE and detected by immunoblotting using 1:10,000 dilution of anti-FLAG M2 monoclonal antibody. L, molecular mass protein ladder. (b) Complementation of colanic acid production in *E. coli* XBF1(pWQ499). Bacteria expressing full length WcaJ (pLA3), WcaJ_CysLess_ (pSEF102) or containing the vector control (pBADNTF) were plated on LB agar supplemented with antibiotics, as needed, and 0.2% (w/v) arabinose. Colonies were evaluated for mucoid phenotype after 24 h.

The topological information derived from the dual fusions was refined by SCAM using methoxypolyethylene glycol maleimide (PEG-Mal) labelling^29^,^30^. WcaJ has 7 cysteine residues at positions 37, 126, 129, 232, 294 and 295, which were replaced by alanine to generate a cysteine-less derivative (WcaJ_Cysless_). WcaJ_Cysless_ was expressed in membrane fractions (Fig. 4a) and restored CA production in strain XBF1(pWQ499) (Fig. 4b), demonstrating that the native cysteine residues in the protein are dispensable for function. PEG-Mal forms irreversible thioester bonds with the sulfhydryl groups on cysteine residues in the presence of a water molecule. Reaction of a cysteine with PEG-Mal results in a mass addition of ~5–10 kDa to the protein, which appears as a band shift by immunoblotting.

**Figure 5:**
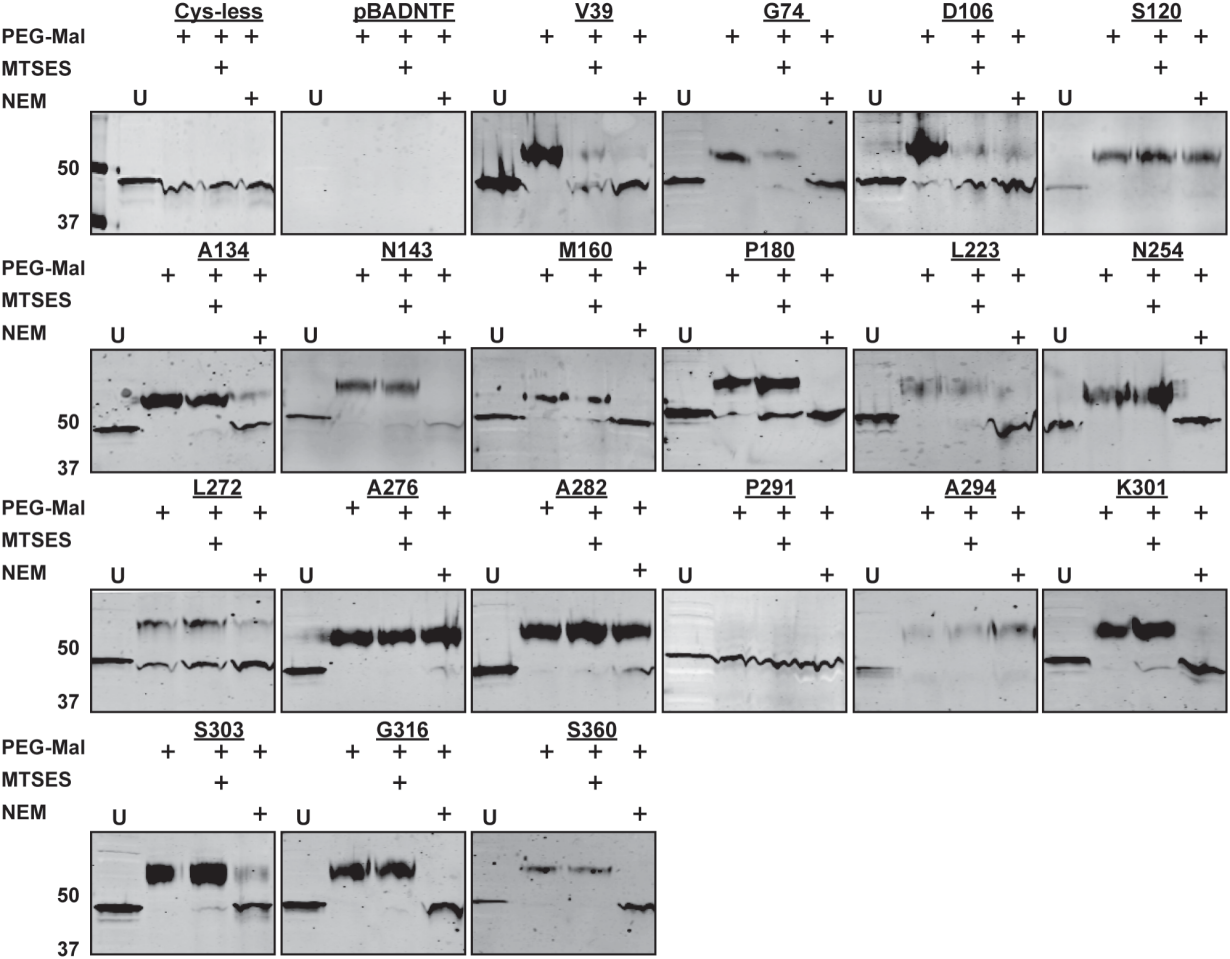
Substituted cysteine labelling of WcaJ_Cysless_ derivatives containing cysteine replacements. Whole bacteria were treated with thiol-blocking reagents MTSES and NEM prior to lysis and labelling with PEG-Mal. The figure is a composite of multiple experiments each involving three experimental samples plus untreated control and the molecular mass markers. All samples were separated on a 14% polyacrylamide gel and detected by 1:10,000 dilution of anti-FLAG M2 monoclonal antibody. The topology of the cysteine residue was determined by the ability of MTSES and NEM to prevent PEG-Mal labelling. PEG-Mal labelling is indicated by a band shift. * and ** indicate the position of WcaJ and PEGylated WcaJ, respectively. U, untreated control.

To determine the topology of the novel cysteine residues in WcaJ, whole cells were pre-treated with thiol-blocking reagents 2-sulfonatoethyl methanethiosulfonate (MTSES; permeable only to the outer membrane, and N-ethylmaleimide (NEM; permeable to both inner and outer membranes). After lysis and sulfhydryl-labelling with PEG-Mal, the topology was assessed by the ability of the thiol-blocking reagents to bind to the introduced novel cysteine residues and prevent them from becoming accessible to PEG-Mal and subsequently causing a band shift. Treatment with both MTSES and NEM prevented labelling of PEG-Mal to residues V39C and D106C indicating that these residues are exposed to the periplasm, corresponding with Pho/LacZ fusion results (Fig. 5). Similarly, residues G74C, A134C, N143C, M160C, P180C, L223C, N254C, K301C, S303C, G316C and S360C could only be labelled in samples pre-treated with MTSES but not with NEM, suggesting they face the cytoplasm (Fig. 5). Labelling of PEG-Mal was observed after treatment with both MTSES and NEM in the reintroduced native cysteine C294 suggesting this residue is in a transmembrane domain. Residue L272C was partially PEGylated after treatment with both MTSES and NEM. This residue is predicted to be on the interface between the cytoplasm and a transmembrane domain, which could explain the partial PEGylation under all treatment conditions. Together, the combined results from reporter fusions and SCAM using PEGylation support a topological model for WcaJ whereby the protein contains two prominent cytosolic regions separated by a single TMH (Fig. 3).

### Analysis of the borders of TMH-V suggests a helix-break-helix structure

**Figure 6:**
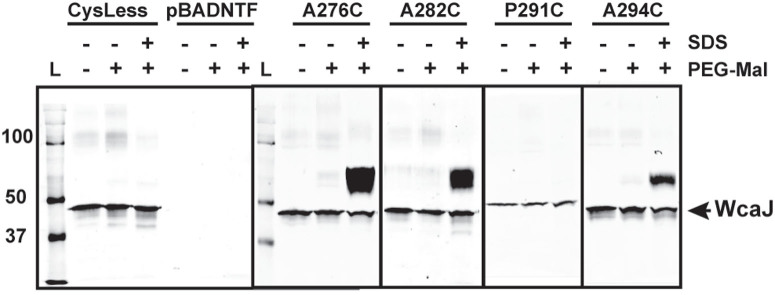
Substituted cysteine labelling in the presence of SDS using PEG-Mal in total membrane preparations expressing _FLAG_WcaJ cysteine replacement proteins. Total membranes were either untreated, incubated with 1 mM PEG-Mal to reveal exposed residues, or 1 mM PEG-Mal with 1% SDS to reveal TMH residues. Samples were examined by SDS-PAGE in 14% polyacrylamide and detected by immunoblotting with 1:10,000 dilution of anti-FLAG M2 monoclonal antibody. Each experiment indicated in the figure was run under identical conditions. Band shift denotes PEGylated polypeptides. Ten μg of protein were labelled, except for WcaJ_P291C_, which required 30 μg. L, molecular mass protein ladder.

Our experimental results indicating that TMH-V separates two cytosolic soluble domains, suggest this TMH has an unusual structure. P291C could not be labelled with PEG-Mal under conditions that allowed PEGylation of nearby residues (Fig. 5). To better examine the behaviour of this P291C replacement mutant, we also performed PEGylation experiments in isolated total membrane protein fractions before and after SDS treatment. This experiment showed that similar to the PEGylation conditions using whole bacteria (Fig. 5), the protein with the P291C replacement could not be labelled, while replacements in the neighbouring residues A276C, A282C and A294C were PEGylated in the presence of SDS (Fig. 6). Together, the results suggest that the cysteine at position 291 is located in a region of the protein adopting a conformation that prevents labelling even after SDS treatment. WcaJ_P291C_ was also poorly expressed and migrated abnormally compared to the other proteins (Figs. 5 and 6), suggesting an alteration of the secondary structure in this mutant protein. However, expression of WcaJ_P291C_ in Δ*wcaJ*(pWQ499) restored colanic acid biosynthesis (Supplemental Figure S3 online), indicating that the replacement of proline by cysteine at this position does not compromise the functionality of WcaJ.

**Figure 7:**
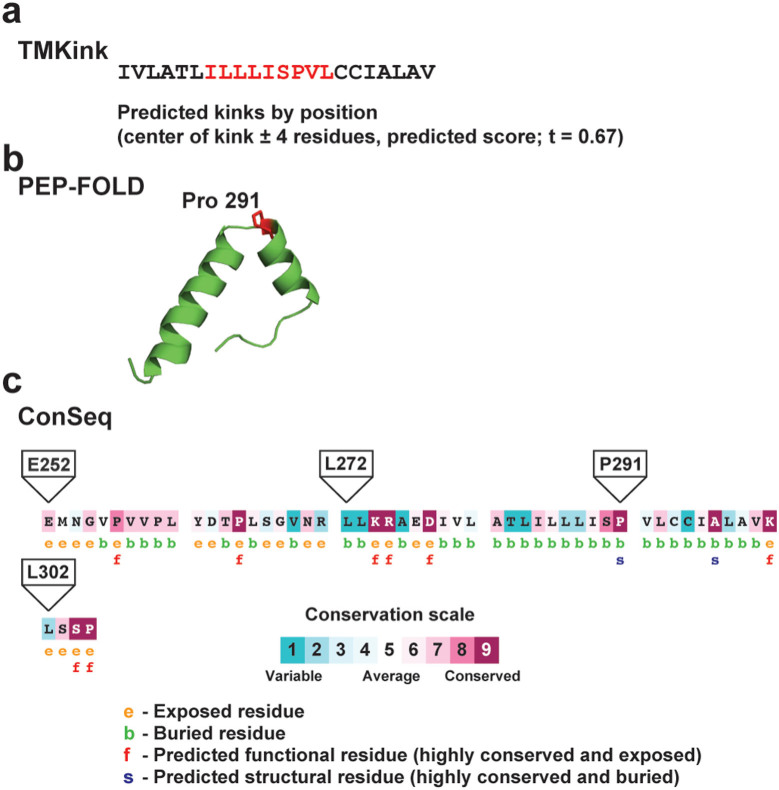
*In silico* analysis of the WcaJ TMH-V. (a) A potential kink (highlighted in red) was identified in the α helix of TMH-V using TMKink. (b) Structural modelling of TMH-V using PEP-FOLD, which predicts a helix-break-helix structure involving serine-290 and proline-291. (c) ConSeq prediction from multiple PHPT homologues identifying conservation scores for each residue and predicting if they are buried or exposed. Proline-291 is a predicted as a highly conserved and buried structural residue. A proline at this position is also conserved in a dataset of PHPT homologues (Supplementary Figure S4 online). The positions of the neighbouring Cys substitutions are also indicated.

To gain additional insight on the potential structure of this TMH, we analysed its secondary structure using PSIPRED^31^, which predicted a helical conformation between L273 and L302. This prediction was in agreement with the experimental boundaries determined for TMH-V. The region encompassing residues R270–G306 was modelled using TMKink, a program specifically tailored to identify kinks within TMHs. Meruelo *et al.*^32^ demonstrated that there are residue preferences in TMHs that contribute to kinks and that a score above the designated threshold indicates a kink in the helix. Using TMKink, we found that R270–G306 contains a predicted kink in TMH-V with a value of 0.719 that it as above the 0.67 cut-off threshold^32^. The predicted kink spans the sequence I284-LLLISPV-L292, which includes P291 (Fig. 7a). Similar results were obtained from TMKink predictions using a dataset of 112 PHPT homologues (Supplementary Table S1 and Supplemental Figure S4 online), obtaining in all cases predicted values higher that the cut-off threshold within a region of the protein that includes a conserved proline at a position corresponding to the P291 residue in WcaJ. We also modelled this region in WcaJ with PEP-FOLD^33^, which revealed a helix-break-helix predicted structure (Fig. 7b). The break region corresponded to S290 and P291. Further analysis using WcaJ residues E252-Y464 with ConSeq^34^ also predicted that this proline is buried (Fig. 7c). PHPT homologues typically fall into two different classes: proteins containing the three canonical domains, (e.g. WbaP and WcaJ), and proteins that have the C-terminal domain including TMH-V (e.g. PssY^5^). Fewer PHPT homologues only have the soluble C-terminal domain, but it is unknown if these proteins are functional. We examined a dataset of 112 PHPT homologues (Supplementary Table S1 online) by Clustal Omega. In most cases, we found a conserved proline residue at the same position corresponding to P291 and within a region in these proteins that is predicted as a transmembrane domain (Supplemental Figure S4 online). Together, our results strongly suggest that TMH-V has a subdomain adopting a helix-break-helix structure and this feature is conserved in PHPT family members.

### Concluding Remarks

This study experimentally elucidates the topology of WcaJ, and also demonstrates that the predicted TMH-V is flanked by two large cytosolic regions, one of which corresponds to the C-terminal domain of the protein. The availability of a cysteine-less WcaJ allowed us to investigate the topology of TMH-V and its neighbouring soluble regions utilizing a substituted cysteine replacement and a sulfhydryl labelling technique with PEG-Mal. These experiments conclusively established that the N- and C-terminal borders of TMH-V (L272 and S304, respectively) face the cytosol while the other intervening residues are buried in the lipid bilayer. P291 was critical to maintain the secondary structure of TMH-V, and alignment of this region with other PHPT members reveals that the proline residue at the equivalent position as P291 is highly conserved. Also, an aspartic acid residue corresponding to that at position 278 in WcaJ that delineates the N-terminal border of TMH-V (Fig. 3) is highly conserved in PHPT proteins (Supplementary Figure S4), suggesting a possible motif including P291 common to the members of this family. Therefore, the results obtained for WcaJ extend to other members of the PHPT family. Studies on the membrane protein caveolin-1 have identified a helix-break-helix structure causing the N- and C-terminal domains of this helix to reside on the same side of the membrane forming an “intra-membrane horseshoe conformation” that does not fully span the membrane^35^. A proline and an isoleucine were among the key residues contributing to the “break” in helix, and their replacement by alanine dramatically disrupted the helix-break-helix structure^35^. Interestingly, the TMH-V is of similar size to the helix-break-helix structure (~28 residues) and has a highly conserved proline and isoleucine residues at positions 291 and 289, respectively. Therefore, the altered migration of WcaJ_P291C_ on SDS-PAGE, its reduced membrane expression or stability, and inaccessibility of the cysteine residue replacing P291 to PEGylation, even after SDS treatment, suggests that TMH-V contains a helix-break-helix structure and cannot span the membrane bilayer, thus explaining why a single predicted TM is found between two regions of the proteins that are facing the cytosol.

In summary, this study reveals an unexpected membrane topology for WcaJ, which is proposed as a signature for all members of the PHPT family. Further investigations are underway in our laboratory to elucidate the function of the central cytosolic domain, previously thought to be located on the periplasmic space.

## Methods

### Bacterial strains, plasmids, media and growth conditions

Bacterial strains and plasmids used in this study are listed in Supplementary Table S2 online. Bacteria were grown at 37°C in Luria-Bertani (LB) medium (Difco) (10 mg tryptone ml^−1^; 5 mg yeast extract ml^−1^; 5 mg NaCl ml^−1^). Growth medium was supplemented with 100 μg ampicillin ml^−1^, 20 μg tetracycline ml^−1^, 40 μg kanamycin ml^−1^ or 30 μg chloramphenicol ml^−1^, as required. The expression of hybrid proteins with β-galactosidase (LacZ) or alkaline phosphatase (PhoA) activities was qualitatively assessed by examining the blue-colony phenotypes on LB plates containing 5-bromo-4-chloro-3-indolyl-β-d-galactopyranoside (XGal) and 5-bromo-4-chloro-3-indolyl-phosphate (XP), respectively, both at a final concentration of 40 μg ml^−1^. XGal and XP were from Boehringer Mannheim. *E. coli* DH5α was transformed with plasmids by the calcium chloride method^36^. *E. coli* CC118 and XBF1 were transformed by electroporation^37^.

### Construction of recombinant plasmids

Primers are listed in Supplementary Table S3 online. DNA ligations were performed with T4 DNA ligase. All constructs were verified by DNA sequencing, which was performed at York University in Toronto, Ontario, Canada, Eurofins MWG Operon, Huntsville, Alabama, USA, or GATC Biotech AG, Constance, Germany. Primers 4779 and 4780 were used to introduce novel histidine residues into the coding region of *wcaJ* gene in pXF1^5^ using *Pfu* AD polymerase (Stratagene). *Dpn*I was added to PCR reactions for overnight digestion of parental plasmid DNA at 37°C. The resulting DNA was introduced into *E. coli* DH5α by transformation and transformants selected on LB-agar containing 100-μg ampicillin ml^−1^. This resulted in pLA1 (Supplementary Table S2 online), which was used as a template to construct pLA3, expressing an N-terminal FLAG fusion to WcaJ (_FLAG_WcaJ), using primers 4911 (containing a *Sma*I restriction site) and 4912 (containing a *Hin*dIII restriction site; Supplementary Table S3 online). The PCR product and the vector pBADNTF were digested with *Sma*I and *Hin*dIII. Digested pBADNTF was also treated with alkaline phosphatase to prevent recircularisation prior to ligation with digested PCR product. After transformation, colony PCR with pBADNTF external primers 252 and 258 (Supplementary Table S3 online) was used to identify the correct construct. Primers 5094 and 5095 served to amplify a region of the *wcaJ* gene encoding the C-terminal domain (WcaJ_E252–Y464_), giving rise to pLA5 (Supplementary Table S2 online).

PhoA fusions to residues S120, N143, M160, P180, and K301 (expressed in plasmids pSEF71, pSEF72, pSEF73, pSEF75, and pSEF74, respectively; Supplementary Table S2 online) were constructed by amplifying the *wcaJ* gene from pLA3 using primer 4911 with primers 5841, 5840, 5839, 5093, 5867, respectively (Supplementary Tables S3 and S4 online). Each PCR product and pAH18 were digested with *Sma*I and *Xba*I, purified and ligated together using T4 ligase. Construction of the A134-PhoA fusion (pLA6, Supplementary Table S2 online) involved amplifying *wcaJ* from pLA3 with primers 4911 and 5258 (Supplementary Tables S3 and S4 online); the PCR product and pSEF74 were digested with *Sma*I and *Xba*I and ligated. For the constructions of the fusions indicated above, PCR amplifications were done with *Pwo* polymerase. *Dpn*I was added to each PCR product to digest the plasmid template and the PCR product was purified using the PCR purification kit (Qiagen) prior to digestion with the restrictions enzymes.

The V39-, G74-, and D106-PhoA fusions (pLA8, pLA9, and pLA7, respectively; Supplementary Table S2 online) were constructed using the direct primer 5257 that amplifies from the *phoA* gene in pLA6 onwards and reverse primers 5364, 5363, and 5259 that amplify from various positions in the *wcaJ* gene (Supplementary Tables S3 and S4 online). The L223-, -N254, and L272-PhoA fusions (pLA18, pLA19, and pLA20, respectively; Supplementary Table S2 online) were also built using the pLA11 DNA template, the direct primer 5257, and reverse primers 5394, 5393, and 5392 (Supplementary Table S3 and S4 online). The WcaJ-LacZ fusions were constructed using the direct primer 5640, which amplifies from the *lacZ* gene in pLA23 onwards, and reverse primers 5364, 5363, 5259, 5841, 5258, 5840, 5839, 5093, 5394, 5393, 5392, 5867, and 5580 (Supplementary Tables S3 and S4 online). Each primer pair mentioned above contained an *Xba*I site. PCR amplifications for these constructs were done with *Pfu* AD polymerase, which extends around the entire plasmid template. *Dpn*I was added to each PCR product to digest the plasmid template and the PCR product was purified as indicated above and digested with *Xba*I. In all of these experiments, ligation products were introduced into *E. coli* DH5α cells by transformation and selected on LB agar supplemented with 100 μg/ml ampicillin.

The cysteine-less _FLAG_WcaJ was constructed by replacing the native cysteine residues with alanine using site directed mutagenesis. Replacements were introduced by PCR using primers containing the desired mutations, and pLA3 as the DNA template (Supplementary Tables S2 and S3). Amplification was performed using *Pfu* Turbo AD polymerase (Stratagene). *Dpn*I was added to PCR reactions for overnight digestion of parental plasmid DNA at 37°C. The resulting DNA was introduced into *E. coli* DH5α by transformation. This resulted in pSEF102 encoding _FLAG_WcaJ_Cys-Less_. Novel cysteine residues were introduced into _FLAG_WcaJ_Cys-Less_ by site directed mutagenesis.

### Complementation of colanic acid biosynthesis in *E. coli* Δ*wcaJ*

Plasmids expressing full length WcaJ (pLA3), WcaJ_CysLess_ (pSEF102), and WcaJ-P291C (pSEF103) were introduced into XBF1/pWQ499 by electroporation and selected on LB-agar supplemented with ampicillin, tetracycline, and kanamycin. Cells were re-plated onto LB-agar with antibiotics with 0.2% arabinose to promote expression of WcaJ. Plates were incubated at 37°C overnight before incubation at room temperature for an additional 24-48 h to observe mucoidity. CA is optimally produced at 20°C^19^.

### Alkaline phosphatase and β-galactosidase assays

The β-galactosidase and alkaline phosphatase activities were quantitated as previously described^38^,^39^.

### Protein analyses

Bacterial cultures containing arabinose-inducible plasmids were grown overnight in LB containing antibiotics, as needed. The next day, cultures were diluted to an optical density measured at 600 nm (OD_600_) of 0.2 in LB with the respective antibiotics. Cultures (100 ml) were grown at 37°C until an OD_600_ of 0.5–0.7 at which point arabinose was added to a final concentration of 0.2% (w/v) and incubation continued for 3 h. Bacteria were harvested by centrifugation 8 000 *g* and cell pellets were frozen at −20°C until needed. Cell pellets were resuspended in 5 ml of 50 mM Tris-HCl, pH 8, with protease inhibitor cocktail (Roche Diagnostics), and lysed at 10 000 PSI with a cell disruptor (Constant Systems Ltd). Cell debris was pelleted at 27 216 *g*. Total membranes were isolated by centrifugation in microfuge tubes at 39 191 *g* and resuspended in 50 μl of 50 mM Tris-HCl, pH 8. Protein concentration was determined by the Bradford protein assay (Bio-Rad). Total membrane preparations were used for immunoblotting and PEGylation. Membrane proteins were separated by 14% SDS-PAGE (For detection of PhoA and LacZ protein fusions 12% and 18% polyacrylamide gels were used; see Supplemental Figures S1 and S2 online). Proteins were transferred to nitrocellulose membrane. Membranes were blocked overnight in 5% Western blocking reagent (Roche Diagnostics) dissolved in TBS pH 7.5 (50 mM Tris-HCl, 150 mM NaCl). The primary antibody, 4.6 mg anti-FLAG M2 monoclonal antibody ml^−1^ (Sigma), was diluted to 1:10 000 and applied for 1.5 h, and the secondary antibody, 2 mg goat anti-mouse Alexa Fluor 680 IgG antibody ml^−1^ (Invitrogen Molecular Probes) was diluted to 1:20 000 and applied for 20 min. Immunoblots were developed using LI-COR Odyssey infrared imaging system. Bio-Rad Precision Plus Protein Standards were used as protein mass standards.

### Protein labelling with methoxypolyethylene glycol maleimide (PEG-Mal)

Plasmids containing _FLAG_WcaJ cysteine substitutions were introduced into *E. coli* XBF1 by CaCl_2_ transformation. Cultures were grown overnight in LB containing antibiotics, as needed. The next day, cultures were diluted to an OD_600_ of 0.2 in LB with the respective antibiotics and grown at 30°C for 1.5 h before induction with 0.2% (w/v) arabinose for 1 h. Cells were adjusted to OD_600_ of 2 in 200 μl of 1× Phosphate Buffered Saline (PBS) (8 mg NaCl ml^−1^, 0.2 mg KCl ml^−1^, 1.44 mg Na_2_HPO_4_ ml^−1^, 0.24 mg KH_2_KPO_4_ ml^−1^, pH7.4) and divided into 50 μl aliquots. Five μl of 55 mM MTSES or NEM in doubled distilled water (ddH_2_O) were added to the samples and incubated at room temperature for 1 h in the dark. Cells were harvested by centrifugation 13,000 rpm for 1 min at 4°C and cell pellets washed with 100 μl of 1× PBS. Cells were resuspended in 22.5 μl of lysis buffer (15 μl of 1 M Tris-HCl pH7.6, 100 μl 10% SDS, 0.376 g urea and up to 1 ml ddH_2_O) and 7.5 μl of 25 mM PEG-Mal solubilised in DMSO was added. Samples were incubated at room temperature for 1 h in the dark before addition of 30 μl of 2× AB buffer (6.84 mM Na_2_HPO_4_, 3.16 mM NaH_2_PO_4_, 50 mM Tris-HCl pH 6.8, 6 M Urea, 1% β-mercaptoethanol, 3% SDS, 10% glycerol, 0.1% Bromophenol Blue) and frozen at −20°C until required. Samples were separated by 14% SDS-PAGE and transferred onto nitrocellulose membranes and immunoblotting performed as indicated above.

### Bioinformatic analyses

Amino acid sequences of PHPT proteins were downloaded from the SYSTERS database (Cluster O 141559; http://systers.molgen.mpg.de/cgi-bin/nph-fetchcluster.pl?CLNR=141559&SIZE1=1&SIZE2=1&LINES=3&v=4)^40^. All these proteins contain PFAM PF02397^41^ corresponding to bacterial sugar transferases. The collection was manually curated to eliminate incomplete sequences, and sequences corresponding to acetyltransferases. The remaining sequences were examined for TMH predictions with TMHMM^13^ and those sequences lacking TMHs were removed from the dataset, which ultimately contained 112 unique sequences (Supplementary Table S1). This dataset is available from the authors as a FASTA file upon request. The PHPT dataset was aligned using Clustal Omega^42^ with default parameters and displayed by Jalview^43^ (Supplementary Figure S4).

## Author Contributions

M.A.V. conceived the study. M.A.V. and S.E.F. wrote the manuscript with contributions from A.F., S.E.F., A.F. and L.A.-R. carried out experimental work. M.A.V. performed the bioinformatics analyses.

## Supplementary Material

Supplementary Information Supplementary Information

## Figures and Tables

**Table 1:** Topological location of selected WcaJ amino acid residues based on reporter fusions and PEGylation

Amino Acid position	Reporter fusion	Plate assay	Miller Units	PEGylation	Location
V39	PhoA	+	2112 ± 95	OUT	PL1
V39	LacZ	−	UD		
G74	PhoA	−	4.0 ± 2	IN	CL1
G74	LacZ	+	0.2 ± 0.1		
D106	PhoA	+	938 ± 70	OUT	PL2
D106	LacZ	−	UD		
S120	PhoA	+	97 ± 2	TM	TMIV
S120	LacZ	+	UD		
A134	PhoA	−	15 ± 1	IN	CL2
A134	LacZ	−	UD		
N143	PhoA	−	15 ± 4	IN	CL2
N143	LacZ	+	1 ± 0.2		
M160	PhoA	−	13 ± 3	IN	CL2
M160	LacZ	+	1 ± 0.2		
P180	PhoA	−	UD	IN	CL2
P180	LacZ	−	0.3 ± 0.1		
L223	PhoA	−	UD	IN	CL2
L223	LacZ	+	65.8 ± 3.2		
N254	PhoA	−	U.D.	IN	CL2
N254	LacZ	+	15 ± 0.6		
L272	PhoA	−	UD	BORDER	CL2
L272	LacZ	+	22 ± 0.4		
A276	ND	ND	ND	TM	TMV
A282	ND	ND	ND	TM	TMV
P291	ND	ND	ND	UD	?
C294	ND	ND	ND	TM	TMV
K301	PhoA	+	611 ± 44	IN	CT
K301	LacZ	+	5 ± 0.5		
S303	ND	ND	ND	BORDER IN	CT
G316	PhoA	−	UD	IN	CT
G316	LacZ	+	13 ± 1		
S360	ND	ND	ND	IN	CT

WcaJ-LacZ/PhoA fusion proteins were expressed in *E. coli* DH5α or CC118 (see Methods). Blue colonies were assessed on plates containing XGal or XP. Alkaline phosphatase and β-galactosidase activities were determined (in triplicate) by measuring the hydrolysis of *p*-nitrophenyl phosphate and ortho-nitrophenyl-β-galactoside, respectively, and the results calculated as Miller units (mean ± standard deviation). PEGylation of WcaJ_Cysless_ with ectopic cysteine residues at the indicated positions was done as described in Methods (see also Fig. 5). UD, undetectable; ND, not done; ?, location could not be established. PL, periplasmic loop; CL, cytoplasmic loop; CT, C-terminal domain.
