# Supplementary material for: Topological analysis of the Escherichia coli WcaJ protein reveals a new conserved configuration for the polyisoprenyl-phosphate hexose-1-phosphate transferase family
Source: Sci Rep. 2015 Mar 17;5:9178. doi: 10.1038/srep09178 (PMC4361858; doi:10.1038/srep09178)
Supplement: Supplementary Information [file srep09178-s1.pdf]

SUPPLEMENTARY INFORMATION

Topological analysis of the *Escherichia coli* WcaJ protein reveals a new conserved configuration for the polyisoprenyl-phosphate hexose-1-phosphate transferase family

Sarah E. Furlong[1], Amy Ford[2], Lorena Albarnez-Rodriguez[1], and Miguel A. Valvano[1,2]*

[1] Centre for Human Immunology, Department of Microbiology and Immunology, University of Western Ontario, London, Ontario, N6A 5C1, Canada
[2] Centre for Infection and Immunity, Queen's University Belfast, Belfast, United Kingdom, BT9 7AE
Correspondence and requests for materials should be addressed to M.A.V (m.valvano@qub.ac.uk)

Table S1 | PHPT homologues used for bioinformatic analyses

**Sequence ID**	**Protein name**	**Number of amino acids**	**Species**
P14186	EXOY_RHISN	226	*Rhizobium* sp. strain NGR234
Q48460	YC14_KLEPN	465	*Klebsiella pneumoniae*
Q57491	Y872_HAEIN	471	*Haemophilus influenzae*
O32274	TuaA protein	179	*Bacillus subtilis*
O34301		193	*Rhizobium etli*
O50321		442	*Erysipelothrix rhusiopathiae*
O85804	CpsA	476	*Erwinia stewartii*
O87932	CpsE	227	*Streptococcus thermophilus*
P71062	YvvC	202	*Bacillus subtilis*
P74057		478	*Synechocystis* sp. strain PCC 6803
P74347		243	*Synechocystis sp.* strain PCC 6803
Q44576		532	*Acetobacter xylinus*
Q45382	WlbG	197	*Bordetella pertussis*
Q54521		455	*Streptococcus pneumoniae*
Q55999		470	*Sphingomonas* sp.
Q56624		186	*Vibrio cholerae*
Q8D3V1		466	*Vibrio vulnificus*
Q8D9C8		213	*Vibrio vulnificus*
Q8DAA6		471	*Vibrio vulnificus*
Q8DL71	Tlr0626 protein	472	*Synechococcus elongatus*
Q8DNL5		243	*Streptococcus pneumoniae* strain ATCC BAA-255 / R6
Q8EMF5		215	*Oceanobacillus iheyensis*
Q8F399		473	*Leptospira interrogans*
Q8F913		462	*Leptospira interrogans*
Q8FG28		464	*Escherichia coli* O6
Q8G1D7		512	*Brucella suis*
Q8G7L5		573	*Bifidobacterium longum*
Q8G7L8	CpsD	514	*Bifidobacterium longum*
Q8GIC9	EpsB	457	*Methylobacillus* sp. 12S
Q8GPA7	Eps7E	462	*Streptococcus thermophilus*
Q8KH81		341	*Aeromonas hydrophila*
Q8KN16		492	*Streptomyces* sp. 139
Q8KW98	RB144	202	*Ruegeria* sp. PR1b
Q8NTF8		204	*Corynebacterium glutamicum*
Q8NYP1	Cap8M	185	*Staphylococcus aureus* strain MW2
Q8NYR9	MW0103 protein	230	*Staphylococcus aureus* strain MW2
Q8P7Z9	GumD	484	*Xanthomonas campestris* pv. *campestris*
Q8PRC0		469	*Xanthomonas axonopodis* pv. *citri*
Q8R6F8		230	*Fusobacterium nucleatum* subsp. *nucleatum*
Q8RBY9		201	*Thermoanaerobacter tengcongensis*
Q8RSV5		469	*Leptothrix cholodnii*
Q8UAP6		294	*Agrobacterium tumefaciens* strain C58 / ATCC 33970
Q8UG08		522	*Agrobacterium tumefaciens* strain C58 / ATCC 33970
Q8UJ36		252	*Agrobacterium tumefaciens* strain C58 / ATCC 33970
Q8VP01		199	*Bordetella avium*
Q8VST2	CpsE	449	*Streptococcus agalactiae*
Q8VTZ3		421	*Leptospira interrogans*
Q8VVI1		477	*Erwinia pyrifoliae*
Q8XMS4		220	*Clostridium perfringens*
Q8XN45		222	*Clostridium perfringens*
Q8XN65		205	*Clostridium perfringens*
Q8XR53		203	*Ralstonia solanacearum*
Q8YMV3	Alr4823	235	*Anabaena* sp. strain PCC 7120
Q8YN51	All4719	243	*Anabaena* sp. strain PCC 7120
Q8YNY9		445	*Anabaena* sp. strain PCC 7120
Q8YPN3	All4160	351	*Anabaena* sp. strain PCC 7120
Q8YT72	All2854	469	*Anabaena* sp. strain PCC 7120

Q8YUQ0		333	*Anabaena* sp. strain PCC 7120
Q8Z5J2	WbaP	476	*Salmonella typhi*
Q92QS6		520	*Rhizobium meliloti*
Q92VP9		216	*Rhizobium meliloti*
Q936H1		185	*Staphylococcus aureus*
Q93CQ5	PssA	263	*Rhizobium leguminosarum* biovar *trifolii*
Q93I52		469	*Sphaerotilus natans*
Q93QV6		198	*Bacteroides fragilis*
Q93TW2	PglB2	525	*Neisseria meningitidis*
Q97EN5		222	*Clostridium acetobutylicum*
Q97EP9		223	*Clostridium acetobutylicum*
Q97GN6		461	*Clostridium acetobutylicum*
Q982Y9		462	*Rhizobium loti*
Q985Q8		184	*Rhizobium loti*
Q988F1		205	*Rhizobium loti*
Q98C89	ExoY	225	*Rhizobium loti*
Q9A5M2	CC2425	512	*Caulobacter crescentus*
Q9A5R3		188	*Caulobacter crescentus*
Q9A878		186	*Caulobacter crescentus*
Q9ABR0		267	*Caulobacter crescentus*
Q9AEE9		421	*Leptospira interrogans*
Q9AHD1	WciI/Cps4E	211	*Streptococcus pneumoniae*
Q9CM33	RfbP	200	*Pasteurella multocida*
Q9EYG6		471	*Actinobacillus pleuropneumoniae*
Q9F0C3	EpsE	219	*Lactobacillus delbrueckii* subsp. *bulgaricus*
Q9F742	WbgY	196	*Plesiomonas shigelloides*
Q9FDJ5		466	*Lactobacillus rhamnosus*
Q9HSZ7	Vng0011c	477	*Halobacterium* sp. strain NRC-1
Q9I1N8		478	*Pseudomonas aeruginosa*
Q9JRN2		472	*Actinobacillus actinomycetemcomitans*
Q9JRR6		400	*Actinobacillus actinomycetemcomitans*
Q9K6L3		207	*Bacillus halodurans*
Q9KTG8		465	*Vibrio cholerae*
Q9KV93		184	*Vibrio cholerae*
Q9KYG5		484	*Streptomyces coelicolor*
Q9PAX8	GumD	484	*Xylella fastidiosa*
Q9PNH0		200	*Campylobacter jejuni*
Q9R927	Cps23fE	455	*Streptococcus pneumoniae*
Q9RFX2	Cps7F	232	*Streptococcus suis*
Q9RG41	Cps9F	200	*Streptococcus suis*
Q9RGI8		202	*Bacteroides fragilis*
Q9RJK7		491	*Streptomyces coelicolor*
Q9RMC7		203	*Acinetobacter lwoffii*
Q9RR58	PglB	413	*Neisseria meningitidis* serogroup B
Q9RZC0		481	*Deinococcus radiodurans*
Q9S4G7		421	*Leptospira interrogans*
Q9WZA0		408	*Thermotoga maritima*
Q9X484		251	*Lactococcus lactis* subsp. *cremoris*
Q9X490		228	*Lactococcus lactis* subsp. *cremoris*)
Q9X4C0	WbaP	476	*Escherichia coli*
Q9X4U9	Cps2E	459	*Streptococcus suis*
Q9X648		485	*Aeromonas hydrophila*
Q9XDP9		452	*Actinobacillus actinomycetemcomitans*
Q9ZAY8		253	*Anabaena* sp. strain CA / ATCC 33047

The dataset is available from the authors as a FASTA file upon request.

Table S2 | List of strains and plasmids

**Strain or Plasmid**	**Relevant Properties**	**Source or Reference**
**Strains**		
CC118	Δ(*ara leu*) Δ*lac phoA galE galK thi rpsL rpsB argE recA*	Laboratory stock
DH5α	F$^-$ φ80*lacZ*ΔM15 *endA recA hsdR*(r$_K^-$ m$_K^-$) *nupG thi glnV deoR gyrA relA1 Δ*(*lacZYA-argF*)*U169*	Laboratory stock
W3110	*rph-1* IN(*rrnD-rrnE*)1, *wbbL*::IS*5*	Laboratory stock
XBF1	W3110, Δ*wcaJ*::*aph*, KnR	[1]
**Plasmids**		
pAF06	pBAD expressing $_{FLAG}$WcaJ-Cys-Less-V39C	This study
pAF07	pBAD expressing $_{FLAG}$WcaJ-Cys-Less-G74C	This study
pAF08	pBAD expressing $_{FLAG}$WcaJ-Cys-Less-D106C	This study
pAF09	pBAD expressing $_{FLAG}$WcaJ-Cys-Less-A134C	This study
pAF10	pBAD expressing $_{FLAG}$WcaJ-Cys-Less-P180C	This study
pAF11	pBAD expressing $_{FLAG}$WcaJ-Cys-Less-L223C	This study
pAF12	pBAD expressing $_{FLAG}$WcaJ-Cys-Less-N254C	This study
pAF13	pBAD expressing $_{FLAG}$WcaJ-Cys-Less-L272C	This study
pAF14	pBAD expressing $_{FLAG}$WcaJ-Cys-Less-K301	This study
pAF15	pBAD expressing $_{FLAG}$WcaJ-Cys-Less-G316C	This study
pAF16	pBAD expressing $_{FLAG}$WcaJ-Cys-Less-S360C	This study
pAF31	pBAD expressing $_{FLAG}$WcaJ-Cys-Less-S120C	This study
pAF32	pBAD expressing $_{FLAG}$WcaJ-Cys-Less-N143C	This study
pAF33	pBAD expressing $_{FLAG}$WcaJ-Cys-Less-M160C	This study
pAH18	pBAD vector expressing $_{FLAG}$PhoA	[2]
pBAD24	Cloning vector, inducible by arabinose, Amp^R	[3]
pBADNTF	pBAD24 vector for N-terminal FLAG fusions, Amp^R	[4]
pLA1	pBAD expressing His5-WcaJ (full length)	This study
pLA3	pBAD expressing $_{FLAG}$WcaJ (full length)	This study
pLA5	pBAD expressing $_{FLAG}$WcaJ$_{E252\text{-}Y464}$ (C-terminal)	This study
pLA6	pBAD expressing $_{FLAG}$WcaJ-A134-PhoA	This study
pLA7	pBAD expressing $_{FLAG}$WcaJ-D106-PhoA	This study
pLA8	pBAD expressing $_{FLAG}$WcaJ-V39-PhoA	This study
pLA9	pBAD expressing $_{FLAG}$WcaJ-G74-PhoA	This study
pLA11	pBAD expressing $_{FLAG}$WcaJ-S360-PhoA	This study
pLA18	pBAD expressing $_{FLAG}$WcaJ-L223-PhoA	This study
pLA19	pBAD expressing $_{FLAG}$WcaJ-N254-PhoA	This study
pLA20	pBAD expressing $_{FLAG}$WcaJ-L272-PhoA	This study
pLA23	pBAD expressing $_{FLAG}$WcaJ-G316-LacZ	This study
pLA24	pBAD expressing $_{FLAG}$WcaJ-G316-PhoA	This study
pLA30	pBAD expressing $_{FLAG}$WcaJ-P180-LacZ	This study
pLA31	pBAD expressing $_{FLAG}$WcaJ-A134-LacZ	This study
pLA32	pBAD expressing $_{FLAG}$WcaJ-D106-LacZ	This study
pLA33	pBAD expressing $_{FLAG}$WcaJ-V39-LacZ	This study
pSEF62	pBAD expressing $_{FLAG}$WcaJ-G74-LacZ	This study
pSEF63	pBAD expressing $_{FLAG}$WcaJ-L272-LacZ	This study
pSEF64	pBAD expressing $_{FLAG}$WcaJ-N254-LacZ	This study
pSEF65	pBAD expressing $_{FLAG}$WcaJ-L223-LacZ	This study
pSEF66	pBAD expressing $_{FLAG}$WcaJ-S120-LacZ	This study
pSEF67	pBAD expressing $_{FLAG}$WcaJ-N143-LacZ	This study
pSEF68	pBAD expressing $_{FLAG}$WcaJ-M160-LacZ	This study
pSEF69	pBAD expressing $_{FLAG}$WcaJ-K301-LacZ	This study
pSEF71	pBAD expressing $_{FLAG}$WcaJ-S120-PhoA	This study
pSEF72	pBAD expressing $_{FLAG}$WcaJ-N143-PhoA	This study
pSEF73	pBAD expressing $_{FLAG}$WcaJ-M160-PhoA	This study
pSEF74	pBAD expressing $_{FLAG}$WcaJ-K301-PhoA	This study
pSEF75	pBAD expressing $_{FLAG}$WcaJ-P180-PhoA	This study
pSEF102	pBAD expressing $_{FLAG}$WcaJ-Cys-Less	This study
pSEF103	pBAD expressing $_{FLAG}$WcaJ-Cys-Less-P291C	This study
pSEF104	pBAD expressing $_{FLAG}$WcaJ-Cys-Less-A282C	This study
pSEF105	pBAD expressing $_{FLAG}$WcaJ-Cys-Less-A276C	This study
pSEF106	pBAD expressing $_{FLAG}$WcaJ-Cys-Less-A294C	This study

pSEF107	pBAD expressing $_{FLAG}$WcaJ-Cys-Less-G316C	This study
pSEF108	pBAD expressing $_{FLAG}$WcaJ-Cys-Less-L272C	This study
pSEF109	pBAD expressing $_{FLAG}$WcaJ-Cys-Less-S303C	This study
pSEF110	pBAD expressing $_{FLAG}$WcaJ-Cys-Less-K301C	This study
pWQ499	pKV102 containing *rcsAK30;* TcR	C. Whitfield
pXF1	pBAD24 encoding W3110 WcaJ	[5]

Table S3 | List of primers used for cloning

Primer Name	Primer Number	Sequence (5'-3')
pBAD forward	252	GATTAGCGGATCCTACCTGA
pBAD reverse	258	GACCGCTTCTGCGTTCTGAT
R-His-WcaJ	4779	ATGGTGGTGATGGTGCATCCTTGTTCCTCCATGGTG
F- His-WcaJ	4780	CACCATCATCACCATACAAATCTAAAAAAGCGC
F-SmaI-WcaJ	4911	CTAGCCCGGGACAAATCTAAAAAAGCGC
R-HindIII-WcaJ	4912	CTAGAAGCTTTCAATATGCCGCTTTGTTA
F-PstI-WcaJCT	5094	CCTGCTGCAGGAGATGAACGGCGTACCG
R-PstI-WcaJCT	5095	TCGACTGCAGCTTGTCATCGTCATCCTTG
F-XbaI-PhoA	5257	CCCGTCTAGAGTCGACCTGCAGCCTGTTCTG
R-XbaI-V39	5364	CGCATCTAGAGACTTCGCAAACCAGCCATAG
R-XbaI-G74	5363	CGCATCTAGAACCGCGCCATGAGCGATAAAA
R-XbaI-D106	5259	CGCATCTAGAGTCGAAATCATTGTTGAACGC
R-XbaI-S120	5841	CGCATCTAGAGCTGGTCAGCGCATACCACGCCA
R-XbaI-A134	5258	CGCATCTAGACGCCCCAATGCGAATACACGA
R-XbaI-N143	5840	CGCATCTAGAGTTATAGCCATGATTACGCAGCC
R-XbaI-M160	5839	CGCATCTAGACATCAGCATTTGCCCGGCGGCTA
R-XbaI-P180	5093	CGCATCTAGACGGGTCGTGGTAAACGCCCAC
R-XbaI-L223	5394	CGCATCTAGAGACCAGTTTTTCACTCGCGCGC
R-XbaI-N254	5393	CGCATCTAGAGCCGTTCATCTCTTCGAGGCGTGA
R-XbaI-L272	5392	CGCATCTAGACAGGCGGTTAACCCCGGAAAG
R-XbaI-K301	5867	CGCATCTAGATTTCACCACCAGCGCAATACAGC
R-XbaI-G316	5580	CGCATCTAGAGCCGTAGCGAGTCTGGCGG

Table S4 | List of primer pairs and plasmid templates used for plasmid constructions.

Plasmid	Primer Pair	Template Plasmid
pLA1	4779/4780	pXF1
pLA3	4911/4912	pLA1
pLA5	5094/5095	pLA3
pLA8 (V39-PhoA)	5257/5364	pLA6
pLA9 (G74-PhoA)	5257/5363	pLA6
pLA7 (D106-PhoA)	5257/5259	pLA7
pSEF71 (S120-PhoA)	4911/5841	pLA3
pLA6 (A134-PhoA)	4911/5258	pLA3
pSEF72 (N143-PhoA)	4911/5840	pLA3
pSEF73 (M160-PhoA)	4911/5839	pLA3
pSEF75 (P180-PhoA)	4911/5093	pLA3
pLA18 (L223-PhoA)	5257/5394	pLA11
pLA19 (N254-PhoA)	5257/5393	pLA11
pLA20 (L272-PhoA)	5257/5392	pLA11
pSEF74 (K301-PhoA)	4911/5867	pLA3
pLA24 (G316-PhoA)	5257/5580	pLA11
pLA33 (V39-LacZ)	5640/5364	pLA23
pSEF62 (G74-LacZ)	5640/5363	pLA23
pLA32 (D106-LacZ)	5640/5259	pLA23
pSEF66 (S120-LacZ)	5640/5841	pLA23
pLA31 (A134-LacZ)	5640/5258	pLA23
pSEF67 (N143-LacZ)	5640/5840	pLA23
pSEF68 (M160-LacZ)	5640/5839	pLA23
pLA30 (P180-LacZ)	5640/5093	pLA23
pSEF65 (L223-LacZ)	5640/5394	pLA23
pSEF64 (N254-LacZ)	5640/5393	pLA23
pSEF63 (L272-LacZ)	5640/5392	pLA23
pSEF69 (K301-LacZ)	5640/5867	pLA23
pLA23 (G316-LacZ)	5640/5580	pLA23

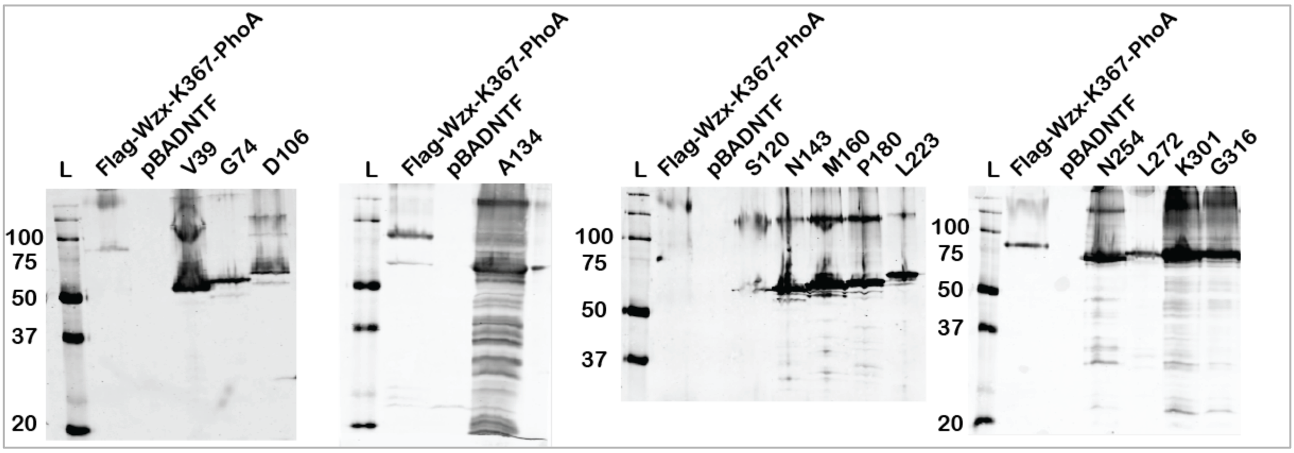

Figure S1. Membrane expression of $_{FLAG}$WcaJ-PhoA fusion proteins in CC118 cells. 30 µg of total membrane preparations from CC118 expressing $_{FLAG}$WcaJ-PhoA fusions were separated by 12% SDS-PAGE and immunoblotting was performed using anti-Flag antibodies. $_{FLAG}$Wzx-K367-PhoA [2] was used as positive control for PhoA western blots and experiments.

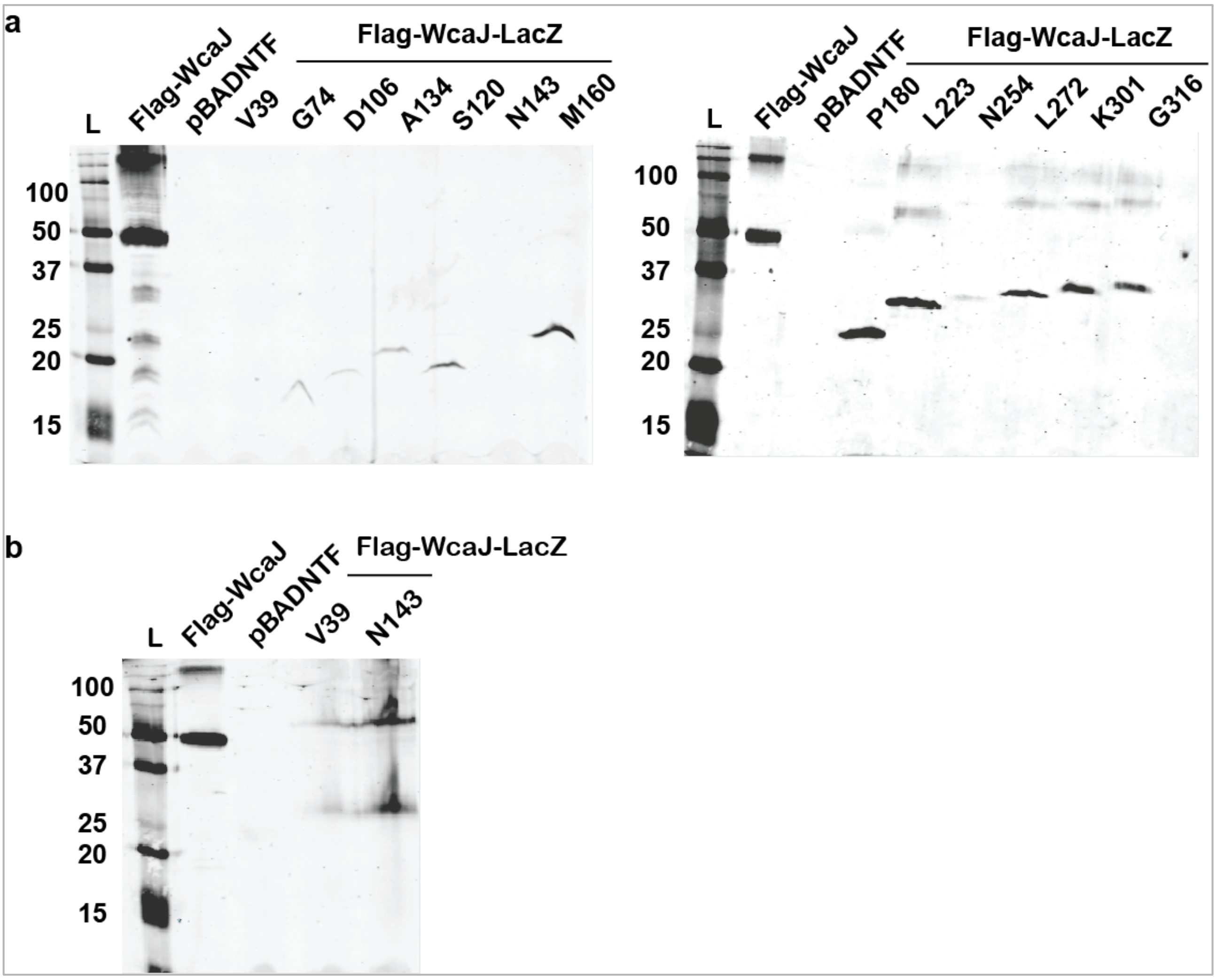

Figure S2. Membrane expression of $_{FLAG}$WcaJ-LacZ fusion proteins in DH5α cells. Total membrane preparations were separated by 18% SDS-PAGE and immunoblotting was performed using anti-FLAG antibodies. (a) 10 µg of total membranes from DH5α expressing $_{FLAG}$WcaJ were used as a positive control. 30 µg of total membranes from DH5α expressing $_{FLAG}$WcaJ-LacZ fusions were separated by SDS-PAGE. (b) 10 µg of total membranes from DH5α expressing $_{FLAG}$WcaJ were used as a positive control. 50 µg of total membranes from DH5α expressing $_{FLAG}$WcaJ-LacZ fusions were separated by SDS-PAGE.

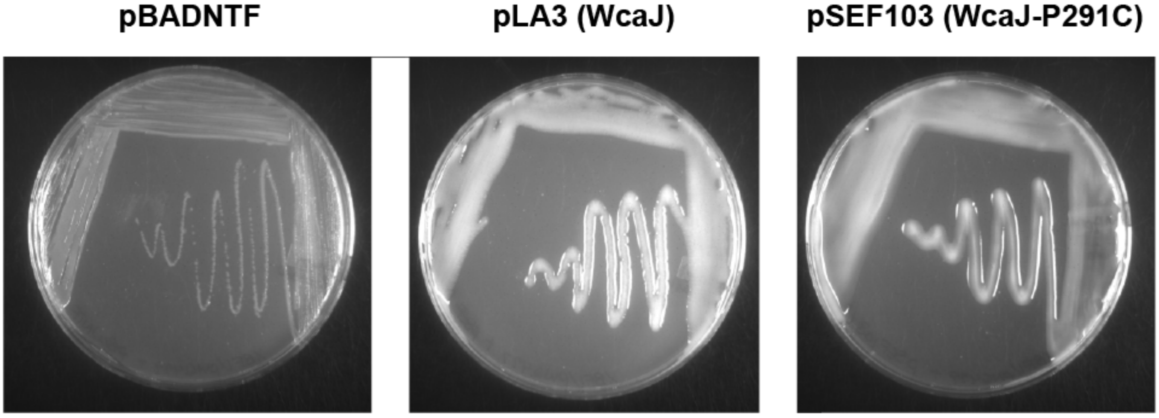

Figure S3. Complementation of colanic acid production by WcaJ$_{P291C}$. The *E. coli* strain Δ*wcaJ*(pWQ499) (XBF1; Table S2) was transformed with plasmids expressing WcaJ, WcaJ-P291C, and the vector control pBADNTF and plates were incubated in LB containing 0.2% arabinose (w/v).

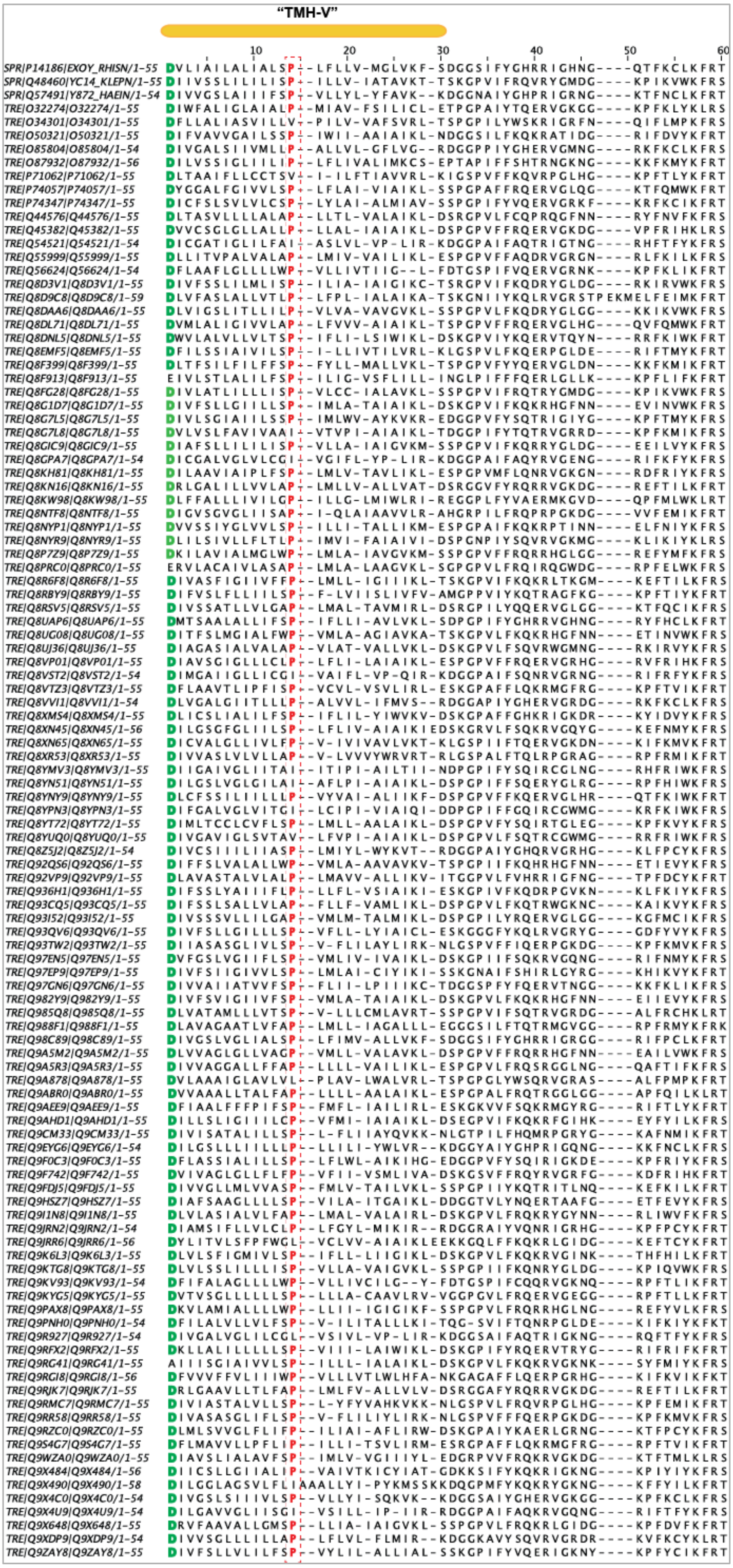
"TMH-V"
10
20
30
40
50
60

Figure S4. Alignment of the TMH-V and C terminal region of PHPT homologues. The dataset is described in Table S3. Initial alignments using Clustal Omega [6] revealed that an aspartic acid residue (in green) corresponding to D-278 in WcaJ is highly conserved (see Fig. 3 in the main manuscript). To facilitate the alignment, all sequences were trimmed to remove all amino acids N-terminally located with the respect to D-278, which serve to mark the predicted border of TMH-V. The highly conserved proline corresponding to P-291 in WcaJ is indicated in red.

Supplementary References

1 Patel, K. B. *et al.* Functional characterization of UDP-Glucose:Undecaprenyl-Phosphate Glucose-1-Phosphate Transferases of *Escherichia coli* and *Caulobacter crescentus*. *J Bacteriol* **194**, 2646-2657. (2012).

2 Marolda, C. L. *et al.* Membrane topology and identification of critical amino acid residues in the Wzx O-antigen translocase from *Escherichia coli* O157:H4. *J. Bacteriol.* **192**, 6160-6171 (2010).

3 Guzman, L. M., Belin, D., Carson, M. J. & Beckwith, J. Tight regulation, modulation, and high-level expression by vectors containing the arabinose pBAD promoter. *J. Bacteriol.* **177**, 4121-4130. (1995).

4 Marolda, C. L., Vicarioli, J. & Valvano, M. A. Wzx proteins involved in O antigen biosynthesis function in association with the first sugar of the O-specific lipopolysaccharide subunit. *Microbiology* **150**, 4095-4105 (2004).

5 Patel, K. B., Ciepichal, E., Swiezewska, E. & Valvano, M. A. The C-terminal domain of the *Salmonella enterica* WbaP (UDP-galactose:Und-P galactose-1-phosphate transferase) is sufficient for catalytic activity and specificity for undecaprenyl monophosphate. *Glycobiology* **22**, 116-122. (2012).

6 Sievers, F. *et al.* Fast, scalable generation of high-quality protein multiple sequence alignments using Clustal Omega. *Molecular Systems Biology* **7:539**, doi:10.1038/msb.2011.75 (2011).
